# Case of Triplets, with an Unusual Shortening of One of the Cords

**Published:** 1856-02

**Authors:** J. Levergood

**Affiliations:** Wrightsville, Pa.


### *Case of Triplets, with an unusual shortening of one of the cords.*

By J. Levergood, M. D., Wrightsville, Pa.

On Monday, Dec. 24th, 1855, I was called to attend Mrs. H., æt. thirty-five, in labor with her fifth child. On an examination per vaginam, the membranes were found ruptured, os uteri fully dilated, and head presenting, and in thirty minutes after my arrival she was delivered of a full-grown healthy child. Upon attempting to apply the usual ligatures to the funis umbilicalis, I found the child so firmly drawn against the mother as to render the application of but one ligature feasible, for the cord was stretched to its utmost capacity, and any endeavor to move the child, for the purpose of applying a second ligature to the cord, would have certainly terminated in its rupture. There being indubitable evidence of the existence of another child in utero, I made an examination to ascertain the nature of the presentation—which was of the breech—when I found, drawn completely into the uterus, the severed cord. After a few expulsive pains, the second child was born; having properly disposed of the two children, and the abdomen being greatly diminished in size, we awaited patiently for the expulsion of the placenta or placentæ, as the case might be, but it not taking place as soon as was desirable, I introduced my hand with the view of extracting it when, *mirabile dictu*, I discovered a third child, head presenting. In a short time it (being still-born) and two placentæ were expelled together. I then turned my attention to the peculiarity which has occasioned this communication, viz.: the exceeding shortness of the umbilical cord belonging to the first child, and found its length to be but *four and a half inches*, including the portion attached to the child.

The above case is, I conceive, an interesting one, in view of the fact that there are upon record but very few well authenticated cases, in which the umbilical cords were as short as in this one. That this unusual shortness of the funis is of very rare occurrence, authors and practical experience abundantly confirm. Churchill, in his admirable treatise on midwifery, states that "the cord varies much; it is *very rarely* less than eight inches, though such cases are on record." Blundell cites a case "in which the cord's length, on measurement, was found not to exceed seven inches." Cazeaux relates a case of a woman whom he delivered with the forceps, "where the cord was only nine inches," and, in a foot note to Churchill's work, we find that the editor, Dr. Huston, calls attention to cases in which the cords were six inches and less in length.

This is the second case of triplets that has happened in this place within the last four years; the first occurred in the practice of my friend, Dr. B. C. Lloyd.

